# In the Catheterization Laboratory, Most Iatrogenic Cardiac Tamponades Require Only Pericardiocentesis: A Single-Center Experience

**DOI:** 10.31083/j.rcm2507237

**Published:** 2024-06-28

**Authors:** Hong Luo, Guangxia Wang, Chunchang Qin, Fengpeng Jia, Xiangsen Shao

**Affiliations:** ^1^Department of Cardiology, The First Affiliated Hospital of Chongqing Medical University, 400042 Chongqing, China

**Keywords:** cardiac intervention, cardiac tamponade, pericardiocentesis, surgery, non-lacerated injury

## Abstract

**Background:**

Cardiac tamponade (CT) is a rare but life-threatening complication of cardiac interventions, requiring immediate pericardial cavity pressure relief. While pericardiocentesis often suffices, and some cases necessitate open-chest surgery. This decision is frequently based on individual physician’s experience. This study aims to identify high-risk CT patients following cardiac intervention, advocating for early, decisive surgical intervention.

**Methods:**

A retrospective analysis was conducted on 51 patients who developed iatrogenic CT at our center between October 2013 and October 2023. Patients were classified based on the necessity for open-chest surgery. The study evaluated a variety of factors, including baseline characteristics, therapeutic approaches, and outcomes.

**Results:**

Of the 51 patients with iatrogenic CT, 49 patients were successfully treated without open-chest surgery, with an average immediate drainage volume of 208.2 ± 173.8 mL. In contrast, the two patients requiring open-chest surgery had significantly higher drainage volumes, exceeding 500 mL, with over 300 mL drained in the first hour, indicating laceration injuries. Patients not requiring open-chest surgery demonstrated favorable outcomes.

**Conclusions:**

The majority of patients with iatrogenic CT and non-lacerated injuries experienced a favorable prognosis following pericardiocentesis. However, in cases of lacerated injuries with drainage volume was above 300 mL per hour, pericardiocentesis alone could not stabilize the hemodynamics due to persistent bleeding. Immediate surgery may be needed in these cases.

## 1. Introduction

Cardiac tamponade (CT) is a critical condition characterized by increased pericardial pressure due to the accumulation of fluid, pus, blood, clots, or gas within the pericardium [1]. This leads to impaired diastolic filling and a subsequent reduction in cardiac output [2]. Common causes include pericarditis, tuberculosis, trauma, tumors, and complications from cardiac interventional procedures [3]. Although infrequent during cardiac intervention, CT is a serious and life-threatening complication with a mortality rate as high as 50% and an incidence rate between 0.089–4.8% [4]. Treatments for CT mainly include pericardiocentesis and pericardiotomy [5]. While guidelines suggest surgery in cases of hemodynamic instability [1, 3], the choice of treatment often relies on the individual clinician’s experience. Our center’s experience suggests immediate pericardiocentesis after CT diagnosis to alleviate pressure in the pericardial cavity.

In this analysis, we sought to assess the conditions under which minimally invasive CT treatments can be used in place of more invasive surgical procedures. We conducted a single-center retrospective study of patients who developed iatrogenic CT over the last ten years. Our results suggest that the mechanism behind myocardial injury and the volume of drainage may be indicators to assess whether or not a patient will need open heart surgery.

## 2. Materials and Methods

### 2.1 Study Participants

This retrospective study analyzed the clinical data of patients with iatrogenic CT in the catheterization laboratory of the First Affiliated Hospital of Chongqing Medical University between 2013 and 2023. We excluded patients whose pericardial effusion did not require pericardiocentesis. Three patients were excluded because the depth of the pericardial effusion in the posterior wall of the left ventricle was less than 5 mm as assessed by echocardiogram and there was only transient deterioration in hemodynamics (systolic blood pressure >90 mmHg) with monitoring of blood pressure every few minutes for several hours. In this cohort, 51 individuals experienced CT during cardiac interventions. Demographic data collected included age, gender, past history of cardiac interventions, echocardiogram, history of anticoagulant and antiplatelet medications, chronic heart failure, existing liver dysfunction, and renal dysfunction. This study also described treatment plans and outcomes after CT, including hemodynamics, drainage volume and occurrence of major adverse cardiovascular events (MACEs) during hospitalization. The ethical approval for this study was granted by the Ethics Review Committee of the First Affiliated Hospital of Chongqing Medical University.

### 2.2 Cardiac Interventional Procedures

In this study, the cardiac interventional procedures included percutaneous coronary intervention (PCI), radiofrequency ablation (RFA), left atrial appendage occlusion (LAAO) and pacemaker implantation.

### 2.3 Definitions of Iatrogenic Cardiac Tamponade and Drainage Volume

Iatrogenic CT is described as a condition in which fluid accumulation in the pericardium as a result of interventional complications resulting in unstable hemodynamics. Immediate drainage volume refers to the amount of pericardial blood that is extracted during pericardiocentesis, immediately following the detection of CT associated with a cardiac intervention. The first-hour drainage volume refers to the volume of pericardial blood drained in the first hour after pericardiocentesis (excluding immediate drainage volume). The second-hour drainage volume refers to the volume of pericardial blood drained in the second hour after pericardiocentesis. Total drainage volume is defined as the total amount of blood drained from the initiation of pericardiocentesis until the removal of the drainage tube during the cardiac intervention.

### 2.4 Diagnosis and Treatment of Iatrogenic Cardiac Tamponade

Diagnosis: (1) The heart is seen as normal or enlarged on X-ray fluoroscopy and shrinks or disappears on inspiration. In addition, a semicircular, translucent band that moves in synchronization with the heart may be detected. (2) Echocardiogram revealing fluid seen as dark areas in the pericardium. (3) The presence of hemodynamic instability.

Treatments: Upon diagnosis of CT during interventional procedures, our center employs a standard treatment protocol that includes volume resuscitation, administration of vasoactive agents, and antagonism of anticoagulants with protamine sulfate. If the coronary artery is fractured or perforated, we utilize a balloon, covered stent, or gelatin to seal the hole. Immediate pericardiocentesis is performed under X-ray or ultrasound guidance. After successful pericardiocentesis, a pigtail catheter is inserted into the pericardial cavity to facilitate continuous drainage. This is followed by the placement of a drainage tube and reinfusion of autologous blood. Key metrics recorded include the immediate drainage volume, the first-hour drainage volume, the second-hour drainage volume and total drainage volume. The pigtail catheter is typically removed 2 days later. If hemodynamics were unstable or bleeding continued, patients were promptly transferred to the operating room for surgical repair.

### 2.5 Definition of Major Adverse Cardiovascular Events

Major adverse cardiovascular events (MACEs) included all-cause mortality, heart failure, cardiac arrest, emergency surgery, cardiac shock, acute myocardial infarction, intervention for ventricular arrhythmias and high-degree atrioventricular block, and non-fatal myocardial infarction.

### 2.6 Data Description

Statistical analysis was conducted using SPSS 26.0 (IBM Corp., Armonk, NY, USA). Quantitative variables were expressed as mean ± standard deviation (SD), while categorical variables were summarized as frequencies and percentages (%).

## 3. Results

### 3.1 Basic Clinical Information and Characteristics of Patients

Over the past decade, our center conducted a total of 51,821 cardiac interventional procedures. Among these, we identified 51 cases of iatrogenic CT related to cardiac interventions. The baseline characteristics of these patients are detailed in Table 1.

**Table 1.** **Basic clinical characteristics of study patients**.

Variables	Cardiac Tamponade (n = 51)
Age (years ± SD)	65.2 ± 13.1
Gender [woman (%)]	26 (51.0%)
BMI (${\mathrm{kg/m}}^{2}$ ± SD)	24.1 ± 4.0
Smoking	25 (49.0%)
Liver dysfunction	4 (7.8%)
Renal dysfunction	6 (11.8%)
Hypertension	25 (49.0%)
Diabetes	8 (15.7%)
Chronic heart failure	9 (17.6%)
Hyperlipidemia	12 (23.5%)
Past history of cardiac intervention	7 (15.7%)
LvEF (% ± SD)	60.1 ± 7.7
Anticoagulant drugs use	8 (15.7%)
Antiplatelet drugs use	10 (19.6%)

BMI, body mass index; LvEF, left ventricular ejection function; SD, standard deviation.

### 3.2 Cardiac Intervention Types

Interventions resulting in CT occurred when patients underwent RFA, PCI, LAAO or pacemaker implantation.

### 3.3 Characteristics of Patients with Cardiac Tamponade

Prior to the diagnosis of CT, 46 patients exhibited clinical symptoms distinct from those without CT, such as chest pain, chest tightness, palpitation, sweating, and emesis. Specifically, among the patients who underwent PCI 7 of 15 (46.7%) reported chest pain or tightness. Among patients undergoing RFA, 10 of 29 (34.5%) patients experienced palpitation.

### 3.4 Treatments of Cardiac Tamponade

All patients identified with CT were diagnosed perioperatively and immediately underwent pericardiocentesis under X-ray fluoroscopy or echocardiography for guidance. The average immediate drainage volume was 221.6 ± 183.3 mL (range 10–1000 mL, Table 2), the first-hour average was 50.7 ± 85.7 mL (range 0–400 mL, Table 2), the second-hour average was 30.2 ± 76.2 mL (range 0–500 mL, Table 2), and the total drainage averaged 471.3 ± 471.1 mL (range 10–2100 mL, Table 2). The immediate mean drainage volume was notably higher in patients who underwent RFA, second only to patients who received LAAO. Following pericardiocentesis, two patients underwent surgical treatment due to continued hemodynamic deterioration, resulting in one fatality. In the remaining patients, hemodynamic stability was successfully restored after pericardiocentesis. Within this group the average systolic and diastolic pressures were 117.2 ± 16.0 mmHg and 70.6 ± 8.0 mmHg, respectively. The pericardial drains were removed within 1–2 days after the procedure. The overall survival rate was 98.0%.

**Table 2.** **Management of cardiac tamponade**.

	All patients (n = 51)	PCI (n = 15)	RFA (n = 29)	Pacemaker implantation (n = 5)	LAAO (n = 2)
Pericardiocentesis	51 (100.0%)	15 (100.0%)	29 (100.0%)	5 (100.0%)	2 (100.0%)
Immediate mean drainage volume (mL ± SD)	221.6 ± 183.3	92.0 ± 43.7	269.1 ± 196.8	204.0 ± 85.0	550.0 ± 70.7
The first-hour mean drainage volume (mL ± SD)	50.8 ± 85.7	40.7 ± 63.2	40.8 ± 64.2	19.0 ± 26.0	350.0 ± 70.7
The second-hour mean drainage volume (mL ± SD)	30.2 ± 76.2	26.7 ± 37.9	21.0 ± 39.1	6.0 ± 13.4	250.0 ± 353.6
Total mean drainage volume (mL ± SD)	471.3 ± 471.1	360.3 ± 380.3	477.9 ± 450.6	283.0 ± 159.1	1680.0 ± 28.3
Rehydration	47 (92.2%)	13 (86.7%)	27 (93.1%)	5 (100.0%)	2 (100.0%)
Vasoactive agents	46 (90.2%)	14 (93.3%)	26 (89.7%)	4 (80.0%)	2 (100.0%)
Protamines	16 (31.4%)	2 (13.3%)	12 (41.4%)	5 (100.0%)	2 (100.0%)
Autologous blood transfusion	16 (31.4%)	1 (6.7%)	13 (44.8%)	1 (20.0%)	1 (50.0%)
Surgery	2 (3.9%)	0 (0.0%)	0 (0.0%)	0 (0.0%)	2 (100.0%)
MACEs	1 (2.0%)	0 (0.0%)	0 (0.0%)	0 (0.0%)	1 (50.0%)

PCI, percutaneous coronary intervention; RFA, radiofrequency ablation; LAAO, left atrial appendage occlusion; MACEs, major adverse cardiovascular events.

### 3.5 Two Cases Received Open-Chest Surgery

Among the 51 cases of iatrogenic CT identified, two were associated with LAAO procedures. These patients presenting an immediate drainage volume exceeding 500 mL. Despite interventions with protamine sulfate and vasopressor therapy, both patients remained in unstable conditions, showing significant ongoing bleeding, as indicated by a first-hour drainage volume surpassing 300 mL. At the time of the surgery, both patients presented with left atrial tears—one with a 3-mm tear and the other with a 5-mm tear—requiring immediate surgical repair.

The postoperative outcomes diverged markedly between the two patients. The individual with the 3-mm tear successfully recovered and was discharged in good health. Conversely, the patient with the 5-mm tear succumbed to multiple organ failure. Further investigation identified the sharp barbs of the left atrial appendage occluder as the precipitating cause of the tears and the subsequent complications, highlighting a critical risk factor associated with the device.

## 4. Discussion

In this study we observed cases of CT with perforation occurred during many procedures, including PCI, RFA, LAAO and the implantation of pacemaker electrodes. The majority of these mild iatrogenic CTs were successfully resolved with pericardiocentesis. Notably, the use of blunt tip instrument did not result in myocardial laceration, preserving myocardial tissue integrity. In patients who only required pericardiocentesis, the mean immediate drainage volume was 208.2 ± 173.84 mL, with a first-hour mean drainage volume of 38.5 ± 60.5 mL. Individuals with myocardial tears and limited bleeding did not require open heart surgery. However, in the distinct cases of two patients with left atrial appendage tears, non-surgical treatment was insufficient to stop the bleeding. While pericardiocentesis provided temporary symptom relief, surgical intervention ultimately became necessary. Surgery may be indicated for individuals exhibiting high drainage volumes to prevent hemodynamic collapse and the potential onset of multi-organ failure.

Over the past decade, our center has performed 51,821 cardiac interventional procedures, with an iatrogenic CT incidence rate of approximately 0.10%. Our center’s experience aligns with the notion that pericardiocentesis suffices as the primary treatment for most iatrogenic CT cases, often precluding the need for open surgery. This is largely because injuries caused by blunt device usually do not disrupt myocardial tissue continuity. This is similar to the finding of Laborderie *et al*. [6], who found that most ventricular pace lead perforations can be resolved by simply withdrawing the catheter. Similarly, Schwerg *et al*. [7] found no significant difference in myocardial thickness between patients with or without perforations caused by leads, with 96% of such perforations being sealed through transvenous lead withdrawal. This was further substantiated by Bauer *et al*. [8] with data from the European heart survey (EHS) PCI Registry, which included over 42,000 patients. They found that among the 124 patients who experienced myocardial perforations, 11.3% developed CT, but only a small percentage (3.3%) required emergency surgery [8]. In a study by Hamaya *et al*. [9], highlighted that only 3.92% of individuals with pericardial tamponade following atrial fibrillation ablation required surgery. Additionally, the need for surgery was attributed to lacerations caused by the left atrial appendage closure device [9]. Furthermore, numerous studies indicate that CT following RFA is manageable with medical interventions alone, without the need for surgery [10, 11, 12].

Iatrogenic CT can occur through different mechanisms depending on the type of cardiac intervention. For PCI, arterial perforations may be caused by the guidewire and arterial tears can result from balloon expansion [13, 14]. Notably, performing RFA for arrhythmias has the potential to injure the atrial and ventricular walls [12]. During the implantation or reposition of pacemaker leads, the electrode may puncture the chamber wall [4, 15]. Specifically, the left atrial appendage is at high risk of injury during the deployment or retrieval of an occluder during LAAO procedures [16]. Given the left atrial appendage’s thin wall, it is particularly susceptible to injury from sharp devices. Moreover, the left atrial appendage’s contraction is less effective at halting the bleeding compared to the atrium and ventricles, increasing the risk of significant hemorrhage.

There are several mechanisms to explain how non-surgical measures could stop the bleeding in patients with iatrogenic CT. (1) Myocardial anatomy and contraction. The inherent anatomical structure of the myocardium, particularly its ability to contract, can naturally prevent further bleeding. However, the efficacy of this mechanism varies with the myocardial thickness and is less effective in the left atrial appendage compared to other cardiac chambers. Additionally, the varying pressure within the cardiac chambers also varies and may contribute to bleeding control. (2) Blunt tip myocardium penetration during pacemaker implantation. The myocardium’s complex three-dimensional myofibril network structure enables spontaneous hemostasis. Circumferentially oriented myofibrils on the epicardial surface that can control the bleeding by fibrillar contraction [17]. For example, right ventricular perforations generated by pacemaker leads usually can be treated with pericardiocentesis alone [18]. (3) PCI-related injury. Arterial elasticity and local thrombus can seal perforations caused during PCI. Temporary balloon inflation may suffice to control the bleeding, and in cases where it does not, a covered stent may be deployed successfully. In our study, one patient received a covered stent to successfully seal the perforation. Al Mawed *et al*. [19] reported a case of delayed CT following PCI, occurring four days post PCI with stent placement, likely due to guidewire perforation. The perforation had initially formed a thrombus, but the thrombus dissolved after resuming anticoagulation, leading to delayed tamponade [19]. (4) Acute CT dynamics. The physiological response to acute CT includes an increased heart rate and shortened diastolic phase, reducing the opportunity for blood ejection from tears. The severity of these tears may vary with the cardiac cycle, limiting the volume of bleeding [20]. For example, in dry pericardiocentesis, right ventricular (RV) myocardial punctures are frequent and most do not require open surgery [21]. This suggests that holes due to direct myocardial puncture may be capable of spontaneous closure.

In certain cases, such as left atrial appendage lacerations, bleeding control through non-operative methods has proven challenging. The primary issue stems not from the puncture wounds themselves but from myocardial lacerations induced by the motion of a sharp needle tip against the beating heart [22]. For example, a 0.3–0.5 mm diameter acupuncture needle may accidentally cause a 2–3 mm tear in the RV wall, which can result in a rapid and significant blood accumulation often beyond the manageability of non-surgical treatment [23, 24, 25]. The resulting lacerated myocardial injuries are frequently followed by hemodynamic instability. Since the injury is transmural, the myocardial contractions cannot seal the hole, and may even exacerbate it, causing the situation to become increasingly critical with each heartbeat. A particularly poignant case reported by Kodikara *et al*. [26] involved a patient who committed suicide using a syringe needle, culminating in CT. Autopsy findings revealed that a 3 cm long syringe needle was used to puncture the left chest, causing two anterior pericardial perforations and laceration of the left anterior descending artery septal branches, illustrating the severe consequences of sharp device-related myocardial injuries [26].

Although pericardiocentesis and surgical intervention have similar prognosis for CT, the potential for increased complications with open surgical drainage necessitates careful patient selection for surgery. Horr *et al*. [27] conducted a comparative analysis of 1281 patients with CT after cardiac interventions, comparing surgical procedures with pericardiocentesis. They concluded that both approaches have similar in-hospital mortality rates (5.3% vs. 4.4%, *p* = 0.49) [27]. Similarly, Saltzman *et al*. [28] found no significant differences in overall mortality and long-term survival rates between the two therapies, but noted that open surgical drainage could introduce additional complications. Wu *et al*. [29] reported CT resulting from atrial fibrillation ablation, and found that if hemodynamic deterioration continues under pericardiocentesis and bleeding persists, surgical repair should be considered.

There are several limitations with this study. First, is the nature of the analysis. Being a retrospective analysis conducted at a single center, the data could be influenced by measurements, operators and techniques, and selection bias. These factors might affect the generalizability of our results to other settings or populations. Second, is the limited sample size. Only 51 cases identified for inclusion, and merely two of them proceeded to open surgery. This limitation underscores the need for future studies with larger sample sizes and a broader array of patients undergoing various percutaneous interventions to confirm our findings. Third, is follow-up duration. Although most patients experienced symptom relief within 1–2 days, our study lacks long-term follow-up data. The absence of extended monitoring may overlook potential late-onset complications or outcomes following the initial treatment period. Finally is the diagnosis of CT etiologies. For the majority of patients, the causes of cardiac tamponade were deduced theoretically rather than verified through direct examination. This approach may introduce uncertainties regarding the precise etiologies of CT in our patient cohort. Future investigations with larger sample sizes, diverse patient groups, and comprehensive long-term follow-up are essential to confirm our observations and address these limitations.

## 5. Conclusions

In patients with cardiac tamponade who undergo immediate pericardiocentesis, the cause of the injury and the amount of drainage volume after tamponade may be used as indicators for the need for emergency surgery. Open surgery may be considered if the wound is lacerated due to a sharp device and when the drainage volume exceeds 300 mL per hour.

## Data Availability

The datasets used and analyzed during the current study are available from the corresponding author on reasonable request.

## Abbreviations

MACEs, major adverse cardiovascular events; PCI, percutaneous coronary intervention; RFA, radiofrequency ablation; LAAO, left atrial appendage occlusion; BMI, body mass index; LvEF, left ventricular ejection function; CT, cardiac tamponade; SD, standard deviation; RV, right ventricular.
